# Complex Interactions Between Circulating Fatty Acid Levels, Desaturase Activities, and the Risk of Gestational Diabetes Mellitus: A Prospective Cohort Study

**DOI:** 10.3389/fnut.2022.919357

**Published:** 2022-07-11

**Authors:** Yue Liu, Yin-Yin Xia, Ting Zhang, Yang Yang, Richard D. Cannon, Toby Mansell, Boris Novakovic, Richard Saffery, Ting-Li Han, Hua Zhang, Philip N. Baker

**Affiliations:** ^1^Department of Obstetrics and Gynaecology, The First Affiliated Hospital of Chongqing Medical University, Chongqing, China; ^2^Department of Occupational and Environmental Hygiene, School of Public Health and Management, Research Center for Medicine and Social Development, Innovation Center for Social Risk Governance in Health, Chongqing Medical University, Chongqing, China; ^3^Mass Spectrometry Center of Maternal Fetal Medicine, Institute of Life Sciences, Chongqing Medical University, Chongqing, China; ^4^Chongqing Key Laboratory of Oral Diseases and Biomedical Sciences, Chongqing Municipal Key Laboratory of Oral Biomedical Engineering of Higher Education, Stomatological Hospital of Chongqing Medical University, Chongqing, China; ^5^Department of Oral Sciences, Sir John Walsh Research Institute, Faculty of Dentistry, University of Otago, Dunedin, New Zealand; ^6^Molecular Immunity, Murdoch Childrens Research Institute, Melbourne, VIC, Australia; ^7^Department of Paediatrics, University of Melbourne, Melbourne, VIC, Australia; ^8^Department of Obstetrics and Gynaecology, The Second Affiliated Hospital of Chongqing Medical University, Chongqing, China; ^9^Institute of Life Sciences, Chongqing Medical University, Chongqing, China; ^10^College of Medicine, Biological Sciences and Psychology, University of Leicester, Leicester, United Kingdom

**Keywords:** gestational diabetes mellitus, fatty acids, arachidonic acid, delta-5 desaturase activity, delta-9–18 desaturase activity

## Abstract

**Objective:**

Maternal abnormal fatty acid desaturation has previously been linked to gestational diabetes mellitus (GDM). However, few studies have investigated this relationship longitudinally throughout pregnancy. In this study, we investigated the relationship between GDM and desaturase activities across the pregnancy trimesters.

**Methods:**

A total of 661 women (GDM = 189, non-GDM = 472) were selected from the Complex Lipids in Mothers and Babies (CLIMB) cohort study. Clinical information and maternal serum were collected at 11–14, 22–28, and 32–34 weeks of gestation. Totally, 20 serum fatty acids were quantified using gas chromatography–mass spectrometry (GC-MS) analysis at each timepoint. Polyunsaturated fatty acid (PUFA) product-to-precursor ratios were used to estimate desaturase and elongase activities including delta-5 desaturase, delta-6 desaturase, stearoyl-CoA desaturase, and elongase.

**Results:**

After adjusting for major potential confounders including maternal age, BMI, primiparity, smoking, and alcohol consumption, we observed a significant increase in the levels of γ-linolenic acid (GLA) and eicosatrienoic acid (DGLA) in the first trimester of women with GDM, whereas GLA and DGLA were reduced in the third trimester, when compared to the non-GDM group. Arachidonic acid (AA) showed an upward trend in the GDM group throughout pregnancy. Estimated delta-6 desaturase and delta-5 desaturase activity were elevated in the first trimester (OR = 1.40, 95% CI 1.03–1.91; OR = 0.56, 95% CI 0.32–0.96) but attenuated in the third trimester (OR = 0.78, 95% CI 0.58–1.07; OR = 2.64, 95% CI 1.46–4.78) in GDM pregnancies, respective to controls. Estimated delta-9–18 desaturase activity (OR = 3.70, 95% CI 1.49–9.19) was increased in women with GDM in later pregnancy.

**Conclusions:**

Our study highlights the potential importance of fatty acid desaturase activities, particularly estimated delta-5 desaturase and delta-9–18 desaturase in the pathophysiology of GDM. These findings may have applications for the early diagnosis and management of GDM.

## Introduction

Gestational diabetes mellitus (GDM) is a pregnancy complication affecting 7−25% of pregnant women globally (1), defined as abnormal glucose tolerance or insulin resistance, with first recognition in pregnancy. Moreover, an epidemiological study of Chinese adults showed that the prevalence of total diabetes continuously increased from 9.7% in 2007 to 11.2% in 2017 (2). GDM is a growing and urgent public health concern that exhibits long-term adverse complications. Furthermore, women with a history of GDM are also at high risk for metabolic syndromes and diseases, including hyperlipidemia, obesity, and diabetes (3–5). GDM is also a strong predictor of type 2 diabetes (T2D) in pregnant women (6), which has been associated with a 40% increase in insulin resistance (7). Therefore, therapeutic intervention during GDM pregnancy may prevent or minimize the occurrence of T2D and other GDM-related complications in later maternal life.

Physiological insulin resistance often occurs during pregnancy, with a 50−60% increase observed in pregnant women (8). Insulin resistance develops in a normal pregnancy to restrict maternal glucose usage and maximize glucose supply to the growing infant (9). In early pregnancy, maternal estrogen, progesterone, and insulin levels are elevated to promote glucose utilization for the biosynthesis of fat and to deposit energy for later gestation (10). Together with elevated energy demand in the mid- and late pregnancy, fat metabolism becomes an essential substitute source of energy for maternal health and fetal development (10). These gestational adaptations lead to physiological hyperlipidemia and elevated levels of free fatty acids (FFAs) in the maternal blood (3). Likewise, several studies showed that polyunsaturated fatty acid (PUFA) metabolism was regulated during pregnancy. Childs et al. (11) reported that liver and plasma docosahexaenoic acid (DHA, n-3) were at higher level, while eicosatrienoic acid (DGLA, n-6) and arachidonic acid (AA, n-6) were at lower level at the end of mice pregnancy. Burdge et al. (12) demonstrated that pregnant mice increased synthesis of hepatic PC16:0/22:6 (1-palmitoyl-2-docosahexaenoyl PC) to supply DHA to fetus. Postle et al. (13) showed that DHA and linoleic acid (LA) in plasma phospholipid were elevated during human pregnancy. In particular, n-6 long-chain PUFAs play an important role in insulin resistance and hyperlipidemia by affecting the production of inflammatory factors and the binding of insulin to its receptor on the cell membrane (14, 15). For instance, AA is an n-6 PUFA that acts as a precursor for the synthesis of pro-inflammatory eicosanoids, including thromboxanes, prostaglandins (PGs), and leukotrienes (LTs) (16). These eicosanoids are known to be involved in regulation of inflammation, insulin resistance, smooth muscle contraction (uterine contraction), platelet aggregation, and blood clotting (17–19). It is well accepted that quantitative and qualitative alternations in the PUFAs, resulting from changes in the level and activity of endogenous desaturase conversions, are associated with many metabolic diseases. For example, low delta-5 desaturase [D5D, AA/DGLA] and high delta-6 desaturase [D6D, γ-linolenic acid/LA] were reported to be related to insulin resistance (20). Stearoyl-CoA desaturase (SCD), including delta-9–18 desaturase [octadecenoic acid/octadecanoic acid], converts saturated fatty acids (SFAs) into monounsaturated fatty acids (MUFAs), which are linked to obesity, insulin resistance, and hypertension (21–23). Most importantly, the increased activity of these enzymes has been associated with the development of T2D and GDM (24–26). Although a number of studies have investigated the cross-sectional association of circulating maternal serum fatty acids with GDM, most have been in relatively small sample sizes (27–30). Moreover, ethnic differences exist in PUFA desaturase activities. Gray et al. (31) demonstrated that D5D activity was relatively higher in African Caribbeans, while D6D activity was significantly lower in Asian Indians in women with previous GDM. Zhao et al. (32) found that only one PUFA, γ-linolenic acid (GLA), was significantly lower in Canadian GDM women at 32–35 gestational weeks. Meanwhile, our previous study showed GLA and other PUFAs were also significantly lower in the Chinese GDM women at 32–34 gestational weeks (33). There is a lack of studies exploring PUFA desaturase activity longitudinally across pregnancy in a pregnant Chinese population.

To improve our current understanding of the pathophysiology of GDM and address the gaps in the literature, this study aimed to prospectively investigate the associations between (1) individual serum PUFAs and (2) fatty acid (FA) desaturase activity, with GDM across pregnancy.

## Methods

### Study Participants

**Figure 1:**
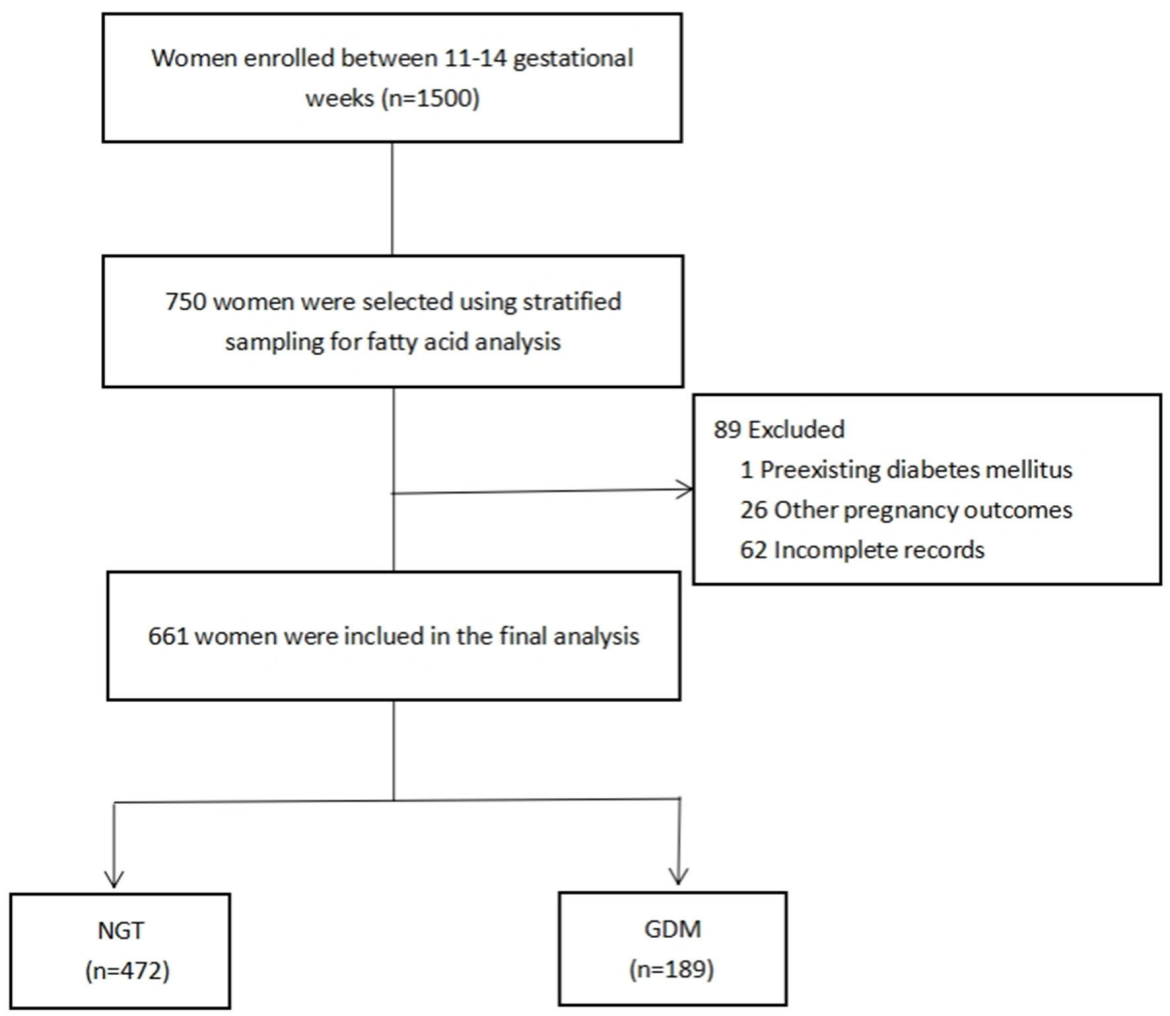
Flowchart of the selection of study participants. NGT, normal glucose tolerance; GDM, gestational diabetes mellitus.

A total of 1,500 pregnant women were recruited from the Complex Lipids in Mothers and Babies (CLIMB) study, a prospective study of pregnant women (Chinese Clinical Trial Register number: ChiCTR-IOR-16007700). The participants were enrolled at prenatal clinics between 11 and 14 gestational weeks between September 2015 and June 2017, at the First Affiliated Hospital of Chongqing Medical University and Chongqing Health Center for Women and Children (34). Of the 1,500 women enrolled, 750 were randomly selected using stratified sampling, by which the proportion of participants per stratum was approximately based on the ratio between the GDM and non-GDM participants (1:3) of the entire cohort. Among them, 89 (one excluded for diabetes mellitus; 26 excluded for pregnancy-induced preterm birth, preeclampsia, or hypertension; and 62 excluded for missing data) were excluded due to incomplete data collection. This led to a total of 661 women in this study, comprising 472 women without GDM and 189 (Figure 1) with GDM. The statistical power to detect the effective sample size of the two groups was calculated from the *post hoc* statistical power of our estimated desaturase activities using GPower software (35). This was diagnosed according to IADPSG guidelines between 22 and 28 weeks of gestation, following at least one of the criteria: fasting blood glucose (FG) ≥5.1 mmol/L, 75 g 1-h oral glucose tolerance test (OGTT) ≥10.0 mmol/L, and 75 g 2-h oral glucose tolerance test ≥8.5 mmol/L. All procedures performed in this study were in full agreement with the International Conference on Harmonization Good Clinical Practice E6 (ICH-GCP) and the Declaration of Helsinki. The study was approved by the Ethics Committee of Chongqing Medical University (No. 2014034), and written informed consent was obtained from all participants.

### Clinical Information and Sample Collection

Maternal clinical characteristics were collected at enrollment, including demographic factors (maternal age, ethnicity, smoking, alcohol consumption, marriage, total years of schooling, primiparity) and gynecologic and obstetric history (gravidity, delivery, miscarriage, infertility). The baseline characteristics of the participants are presented in Table 1. Blood pressure, body mass index (BMI), and overnight fasting maternal blood samples were collected by trained nurses at three visits during pregnancy (11–14, 22–28, and 32–34 weeks of pregnancy). Fasting blood samples were collected from the antecubital vein into SST vacutainer tubes containing separator gel, followed by centrifugation twice (3,000 rpm at 4°C for 10 min, then 4,000 rpm at 4°C for another 10 min). The supernatant was aliquoted into cryotubes (Micronic, Lelystad, The Netherlands) and then stored at −80°C until it was further processed.

**Table 1:** Clinical characteristics of pregnant women with normal glucose tolerance (NGT) and gestational diabetes mellitus (GDM).

	**GDM (*n* = 189)**	**NGT (*n* = 472)**	***p-*value**	***q-*value**
Age (years)	29 (27, 32)	28 (26, 30)	<0.001^***^	<0.001^***^
**BMI (kg/m** ^**2**^ **)**				
1^st^ trimester	21.9 (19.9, 24.0)	20.8 (19.3, 22.8)	<0.001^***^	<0.001^***^
2^nd^ trimester	24.0 (22.0, 26.2)	23.0 (21.2, 25.1)	<0.001^***^	<0.001^***^
3^rd^ trimester	25.6 (23.6, 28.0)	24.6 (22.6, 26.9)	0.001^**^	0.001^**^
Fasting blood glucose (mmol/L)	5.1 (4.7, 5.2)	4.6 (4.4, 4.8)	<0.001^***^	<0.001^***^
1 h postprandial blood glucose (mmol/L)	9.7 (8.4, 10.5)	7.3 (6.4, 8.2)	<0.001^***^	<0.001^***^
2 h postprandial blood glucose (mmol/L)	8.5 (7.5, 9.1)	6.7 (5.9, 7.3)	<0.001^***^	<0.001^***^
**Smoking and drinking**, ***n*** **(%)**				
Yes	0 (0)	4 (0.8)	0.58	0.38
No	189 (100)	468 (99.2)		
**Primiparity**, ***n*** **(%)**				
Yes	129 (68.3)	378 (80.1)	0.002^**^	0.002^**^
No	60 (31.7)	94 (19.9)		
**Miscarriages**, ***n*** **(%)**				
Yes	92 (48.7)	215 (45.6)	0.49	0.37
No	97 (51.3)	257 (54.4)		
**Educational years**	16 (15, 16)	16 (15, 16)	0.45	0.37
**Gestational age at sampling (weeks)**				
1st trimester	12.7 (12.1, 13.4)	12.7 (12.1, 13.3)	0.43	0.37
2nd trimester	24.3 (23.7, 24.7)	24.1 (23.7, 24.7)	0.71	0.42
3rd trimester	32.3 (31.9, 32.9)	32.3 (31.9, 32.7)	0.59	0.38

*The values are represented as median with IQR (interquartile range) or n (%)*.

*p-values are for statistical comparison between the two groups (Mann–Whitney test or chi-square test)*.

*The q-values (adjusted p-values) were performed by the false discovery rate (FDR)*.

*p-values and q-values are significant at p, q < 0.05*;

*** *p, q < 0.001*,

** *p, q < 0.01*.

*BMI, body mass index; NGT, normal glucose tolerance; GDM, gestational diabetes mellitus*.

### Reagents and Fatty Acid Standards

Totally, 21 fatty acid standards were purchased from Sigma-Aldrich (St. Louis, MO, USA; Supplementary Table 5). Methanol (chromatography grade) and n-hexane (chromatography grade) were purchased from Merck-Chemicals, KGaA (Germany). Hydrochloric acid (35%, w/w) and Milli-Q pure water were purchased from Guangzhou Chemical Reagent Factory (Guangzhou, China). Stock solutions (250 mg/ml) of the 20 fatty acids and the internal standard (heptadecanoic acid) were prepared in n-hexane. The working solutions consisted of methanol and were at concentrations of 0.10, 0.50, 2.5, 25, 100, and 250 mg/L. All solutions were stored at −20°C until used. A 20 mg/L heptadecanoic acid n-hexane solution was used as an internal standard working solution.

### Serum Fatty Acid Derivatization and Fatty Acid Methyl Ester (FAME) Extraction

A measure of 250 μl of thawed serum obtained from each participant was mixed with 250 μl of the internal standard solution, followed by the addition of 1 ml hydrochloric acid/methanol and further mixed using a vortex. The prepared sample was then incubated at 90°C for 3 h using an electric blast drying oven (Shanghai Yiheng Scientific Instrument Co., Ltd.). After the sample had cooled, 2 ml of n-hexane was added to recover the fatty acid methyl ester products, and the sample was vortexed. The supernatant was separated after centrifugation (4,000 rpm at 4°C for 10 min) and then evaporated until dry under nitrogen. Following the addition of 400 μl n-hexane to dissolve the methyl esterification product of the fatty acids, the suspension was mixed and then transferred to a sample vial (Agilent Technologies, USA) before mass spectrometry analysis.

### Gas Chromatography–Mass Spectrometry (GC-MS) Analysis

GC-MS analysis was performed using an Agilent 7890B gas chromatograph unit with a 5977A mass spectrometer (Agilent Technologies, USA). The fatty acid separation was carried out on a DB-23 capillary column (0.18 mm × 20 m × 0.20 μm, Agilent Technologies, USA). Helium was selected as the carrier gas, with a constant flow rate of 0.78 ml/min. A measure of 1 μl of derivatized sample was injected into the inlet, which was set to 230°C, using the splitless inlet mode. Free fatty acid methyl esters were isolated using the following oven temperature program: (1) 50°C hold for 1 min; (2) increase to 175°C at 25°C/min; (3) reach 223°C at 4°C/min; and (4) hold at 223°C for 8 min. The ion-trap mass spectrometer was used in selected ion scan (SIM) and full scan (Scan) monitoring modes (mass range: 60.00–450.00 *m*/*z*). The solvent delay time was set at 10 min, the source temperature was set at 220°C, and the transfer line was maintained at 230°C.

### Fatty Acid Quantification and Statistical Analysis

The chromatographic area of all fatty acid peaks was obtained using Agilent ChemStation (version 2.6). Levels of individual serum fatty acids were first normalized to the internal standard and then quantified as absolute concentration using calibration curves derived from the corresponding chemical standard. Desaturase and elongase activities were estimated as fatty acid product-to-precursor ratios and were calculated as follows: D5D [C20:4n-6/C20:3n-6], D6D [C18:3n-6/C18:2n-6], delta-9–16 desaturase [C16:1n-7/C16:0], delta-9–18 desaturase [C18:1n-9/C18:0], and elongase [C22:5n-3/C20:5n-3]. The normality of distribution of the derived variables was evaluated by inspection of data distribution histograms and the results of a Shapiro–Wilk *W*-test. The non-parametric Mann–Whitney *U*-test was used for continuous variables, and the chi-square test for categorical variables when comparing clinical characteristics, as appropriate. The Mann–Whitney *U*-test with a false discovery rate (FDR, *q*-value) test and binary logistic regression were performed to compare fatty acid concentrations and desaturase differences between women with GDM and controls. The logistic regression analysis was adjusted for potential confounders, which included maternal age, smoking, alcohol consumption, primiparity, and BMI. Prior to binary logistic regression analysis, all fatty acids and ratios were ln-transformed for statistical comparisons. A linear mixed model was used to evaluate the interactions between GDM and desaturase activities across the three timepoints, using the lme4 R package (36). Values were expressed as medians (25th percentile, 75th percentile). Values for *q* < 0.05 were considered statistically significant. The *q*-values (adjusted *p*-values) were generated by FDR using the qvalue R package (37). All analyses were performed using SPSS software (version 23.0). Figures were illustrated using the ggplot2 R package (38).

## Results

### Participant Characteristics

The *post hoc* power analysis indicated that the total sample sizes needed to achieve the true difference between the GDM and non-GDM groups for the Wilcoxon–Mann–Whitney test and logistic regression using our studied population were 165 and 210, respectively (Supplementary Figures 1, 2). Compared with women who had normal glucose tolerance (NGT), the women with GDM were significantly different in age (years), primiparity, oral glucose tolerance test (OGTT) outcome, and BMI (*p* < 0.05, *q* < 0.05), as shown in Table 1. Women in the GDM group had significantly higher age than those who were non-GDM; primiparity was significantly lower in the GDM group; women with GDM had a significantly higher OGTT at each trimester than those in the NGT group; BMI at each trimester was significantly higher in the GDM group than in the NGT group. Other maternal characteristics including smoking, alcohol consumption, history of previous miscarriage, educational attainment (years), and gestational age at sampling exhibited no significant differences.

### Differences in the Levels of Serum Fatty Acids Throughout Trimesters

As shown in Supplementary Tables 1–3, maternal serum fatty acids generally increased throughout the course of pregnancy in both GDM and NGT women. In addition, the majority were found to be at higher concentrations in GDM women than in the NGT women at various timepoints. We observed significantly higher concentrations of three PUFAs [α-linolenic acid (ALA, *p* = 0.01, *q* = 0.02), GLA (*p* < 0.001, *q* < 0.001), and DGLA (*p* = 0.001, *q* = 0.007)] in the GDM group in the first trimester; two PUFAs [ALA (*p* = 0.01, *q* = 0.05) and eicosapentaenoic acid (EPA, *p* = 0.03, *q* = 0.07)] in the second trimester; and one PUFA [EPA (*p* = 0.001, *q* = 0.09)] in the third trimester. By contrast, GLA (*p* = 0.04, *q* = 0.09) and DGLA (*p* = 0.02, *q* = 0.09) were found in lower concentrations in the third trimester of women with GDM, while AA showed an upward trend in the concentration level across pregnancy only in the GDM group.

### Longitudinal FA Enzymatic Activity Profiles and Differences in the Levels Across Trimesters

**Figure 2:**
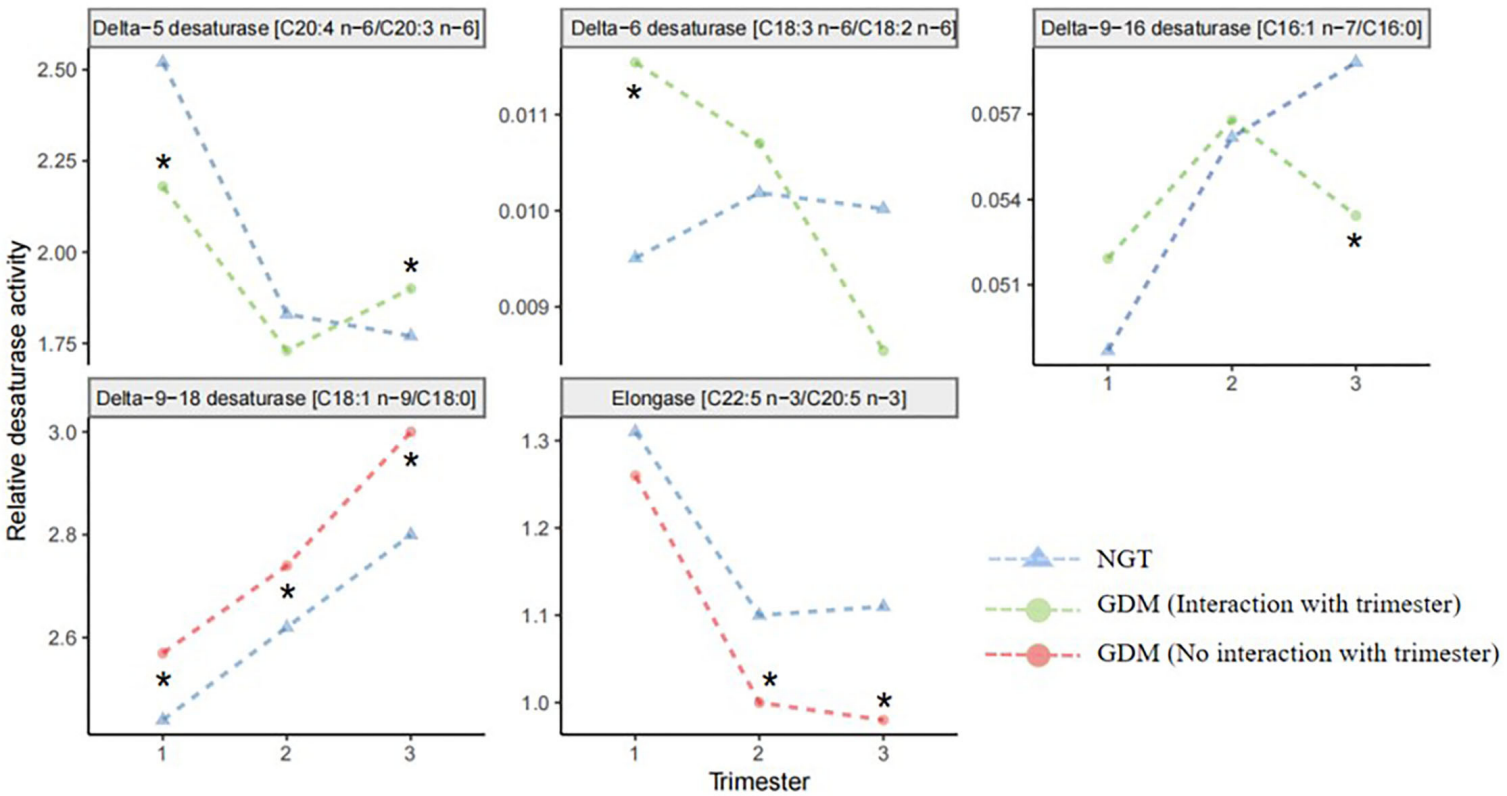
Line plots of the robust fitted effects of GDM across the pregnancy. Circles represent the fitted effects in GDM, and triangles represent NGT. When the line is orange, there is no significant interaction with trimester. When the line is green, the effect changes across the trimester. Fatty acid desaturase activities were entered into the mixed model as the response variable; GDM and trimester were assigned as fixed effects; the individual was assigned as a random effect. NGT, normal glucose tolerance; GDM, gestational diabetes mellitus. *Significant difference between GDM and NGT.

The longitudinal profiles of the five FA enzymatic activities under investigation were quantified from the first to the third trimester (Figure 2) based on fatty acid product-to-precursor ratios between the GDM group and NGT group. These included D5D, D6D, and delta-9–18 desaturase, between the two groups at weeks 11–14, as shown in Table 2. At 22–28 weeks, estimated delta-9–18 desaturase and elongase showed significant differences between groups. At 32–34 weeks, all fatty acid ratios showed a significant difference between GDM and NGT women, except D6D. Furthermore, there were significant interactions between GDM and trimester for the estimated D5D, D6D, and delta-9–16 desaturase activities as calculated using a mixed model with confounders (age, BMI, parity) and without confounders (Supplementary Table 4). The GDM and NGT groups showed an inverse relationship in these desaturase activities between the first and the third trimester, indicating that there were longitudinal differences in these desaturase activities and their relationship with GDM (Figure 2). By contrast, no interaction between GDM and trimester was observed for delta-9–18 desaturase or elongase activities. The GDM and NGT groups showed consistently higher enzymatic activities for delta-9–18 desaturase and elongase across trimesters, respectively.

**Table 2:** Desaturase activities and odds ratios in the first, second, and third trimesters between pregnant women with normal glucose tolerance (NGT) and gestational diabetes mellitus (GDM).

	**GDM (*n* = 189)**	**NGT (*n* = 472)**	***p*-value^**a**^**	***q-*value**	**OR (95%CI)**	***p*-value^**b**^**
**1st trimester**						
Delta-5 desaturase [C20:4 n−6/C20:3 n−6]	2.18 (1.75, 2.97)	2.52 (2.03, 3.03)	0.002^**^	0.004^**^	0.56 (0.32,0.96)	0.04^*^
Delta-6 desaturase [C18:3 n−6/C18:2 n−6]	0.0115 (0.0078, 0.0161)	0.0095 (0.0068, 0.0139)	0.001^**^	0.004^**^	1.40 (1.03,1.91)	0.04^*^
Delta-9–16 desaturase [C16:1 n−7/C16:0]	0.0519 (0.0408, 0.0636)	0.0487 (0.0397, 0.0610)	0.07	0.06	1.42 (0.82,2.45)	0.21
Delta-9–18 desaturase [C18:1 n-9/C18:0]	2.57 (2.23, 2.94)	2.44 (2.16, 2.82)	0.04^*^	0.04^*^	1.90 (0.78,4.60)	0.16
Elongase [C22:5 n-3/C20:5 n-3]	1.26 (0.97, 1.65)	1.31 (0.94, 1.78)	0.31	0.21	0.93 (0.64,1.35)	0.70
∑n-3/n-6	0.12 (0.10, 0.14)	0.11 (0.10, 0.13)	0.01^*^	0.02^*^	1.84 (0.88,3.81)	0.10
**2nd trimester**						
Delta-5 desaturase [C20:4 n−6/C20:3 n−6]	1.73 (1.4, 2.24)	1.83 (1.51, 2.22)	0.52	0.33	0.94 (0.53,1.68)	0.83
Delta-6 desaturase [C18:3 n−6/C18:2 n−6]	0.0107 (0.008, 0.0163)	0.0102 (0.0069, 0.0158)	0.20	0.14	1.22 (0.90,1.65)	0.21
Delta-9–16 desaturase [C16:1 n−7/C16:0]	0.0568 (0.0433, 0.0756)	0.0562 (0.0444, 0.0708)	0.91	0.55	0.99 (0.62,1.58)	0.96
Delta-9–18 desaturase [C18:1 n-9/C18:0]	2.74 (2.42, 3.04)	2.62 (2.29, 3.00)	0.03^*^	0.03^*^	2.24 (0.93, 5.40)	0.07
Elongase [C22:5 n-3/C20:5 n-3]	1.00 (0.71,1.38)	1.10 (0.82, 1.50)	0.02^*^	0.02^*^	0.68 (0.47, 1.00)	0.05
∑n-3/n-6	0.14 (0.11, 0.16)	0.13 (0.11, 0.15)	0.07	0.06	1.82 (0.93, 3.56)	0.08
**3rd trimester**						
Delta-5 desaturase [C20:4 n−6/C20:3 n−6]	1.90 (1.52, 2.38)	1.77 (1.45, 2.14)	0.002^**^	0.004^**^	2.64 (1.46, 4.78)	0.001^**^
Delta-6 desaturase [C18:3 n−6/C18:2 n−6]	0.0085 (0.0065, 0.0133)	0.0100 (0.0068, 0.0144)	0.06	0.05	0.78 (0.58, 1.07)	0.12
Delta-9–16 desaturase [C16:1 n−7/C16:0]	0.0534 (0.0447, 0.0666)	0.0588 (0.0465, 0.0767)	0.01^*^	0.02^*^	0.59 (0.36,0.98)	0.04^*^
Delta-9–18 desaturase [C18:1 n-9/C18:0]	3.00 (2.62, 3.33)	2.80 (2.46, 3.13)	0.001^**^	0.004^**^	3.70 (1.49, 9.19)	0.005^**^
Elongase [C22:5 n-3/C20:5 n-3]	0.98 (0.69, 1.41)	1.11 (0.82, 1.56)	0.002^**^	0.004^**^	0.58 (0.40,0.85)	0.005^**^
∑n-3/n-6	0.14 (0.12, 0.17)	0.13 (0.11, 0.16)	0.003^**^	0.005^**^	2.26 (1.17, 4.35)	0.02^*^

*Data are presented as medians (25th percentile, 75th percentile) for the Mann–Whitney test; the data have been ln-transformed for binary logistic regression*.

*Binary logistic regression analyses were adjusted for age, BMI, smoking and drinking, primiparity*.

a *Values are for statistical comparison between the two groups (Mann–Whitney test)*.

b *Values are for odds ratios between the two groups (binary logistic regression)*.

*The q-values (adjusted p-values) were performed by the false discovery rate (FDR)*.

*p-values and q-values are significant at p, q < 0.05*;

** *p, q < 0.01*,

* *p, q < 0.05*.

*NGT, normal glucose tolerance; GDM, gestational diabetes mellitus; OR, odds ratio; CI, confidence intervals*.

### Adjusted Odds Ratios Between Each Estimated FA Enzymatic Activity and Risk of GDM in the First, Second, and Third Trimesters

**Figure 3:**
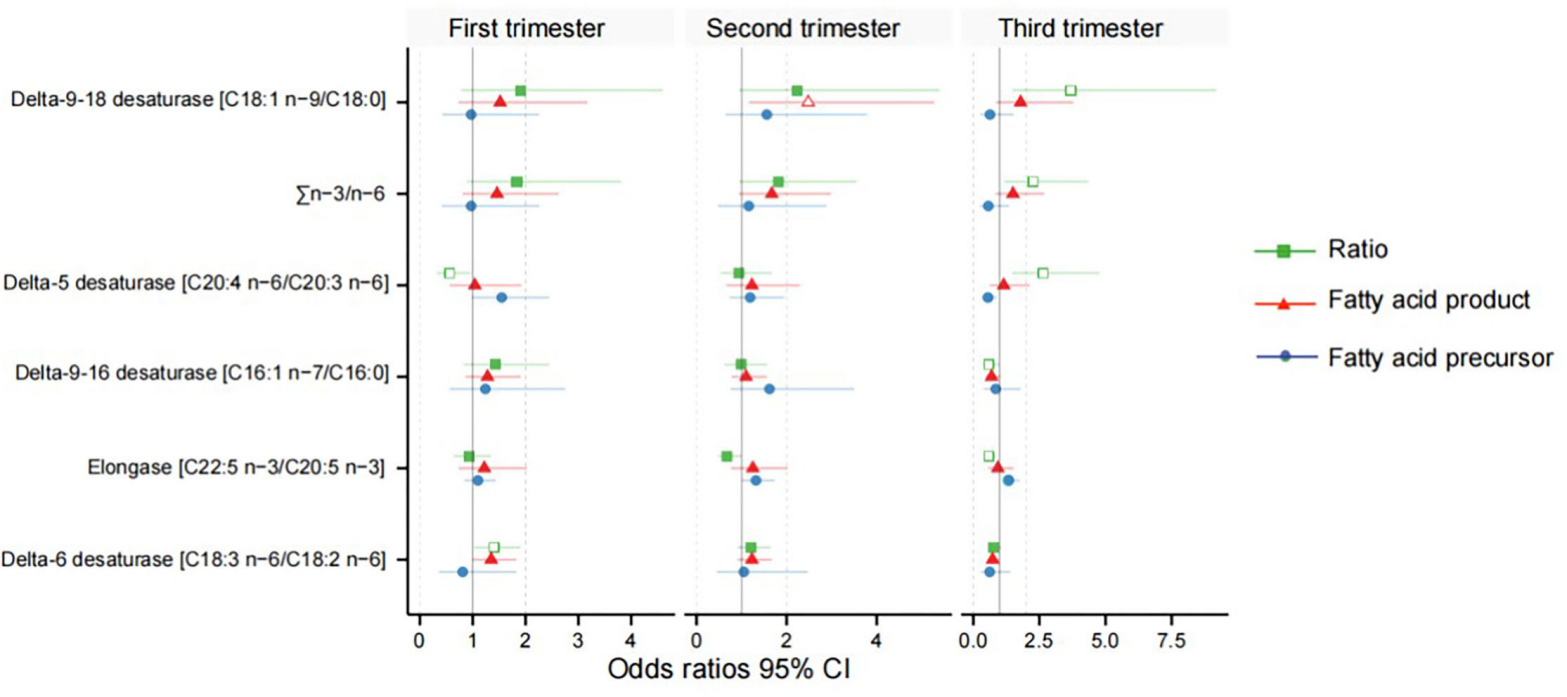
Odds ratios between the desaturase activities and gestational diabetes mellitus. Green indicates the fatty acid product-to-precursor ratios; red indicates the fatty acid product; blue indicates the fatty acid precursor. A hollow shape represents the difference is significant, and solid is non-significant.

After adjusting for major confounders, ALA was found to be significantly higher in women with GDM at weeks 11–14 [odds ratios (OR) = 1.56, 95% CI 1.03–2.36] and 22–28 (OR = 1.69, 95% CI 1.14–2.50; Supplementary Tables 1, 2); EPA (OR = 1.33, 95% CI 1.00–1.76) was also higher in women with GDM at weeks 32–34. However, GLA (OR = 0.73, 95% CI 0.53–1.00) and DGLA (OR = 0.55, 95% CI 0.33–0.90) were significantly lower in women with GDM at weeks 32–34 (Supplementary Table 3). Meanwhile, in women with GDM, estimated D5D activity was lower at 11–14 weeks (OR = 0.56, 95% CI 0.32–0.96) and higher at 32–34 weeks (OR = 2.64, 95% CI 1.46–4.78); estimated D6D activity (OR = 1.40, 95% CI 1.03–1.91) was significantly higher in the serum at weeks 11–14; delta-9–16 desaturase (OR = 0.59, 95% CI 0.36–0.98) and elongase (OR = 0.58, 95% CI 0.40–0.85) were lower in at weeks 32–34; and estimated delta-9–18 desaturase (OR = 3.70, 95% CI 1.49–9.19) and ∑n-3/n-6 (OR = 2.26, 95% CI 1.17–4.35) activities were significantly higher in the women with GDM at weeks 32–34 (Table 2, Figure 3).

### The Changes of Estimated Desaturase Activities in the n-6 Metabolic Pathway

**Figure 4:**
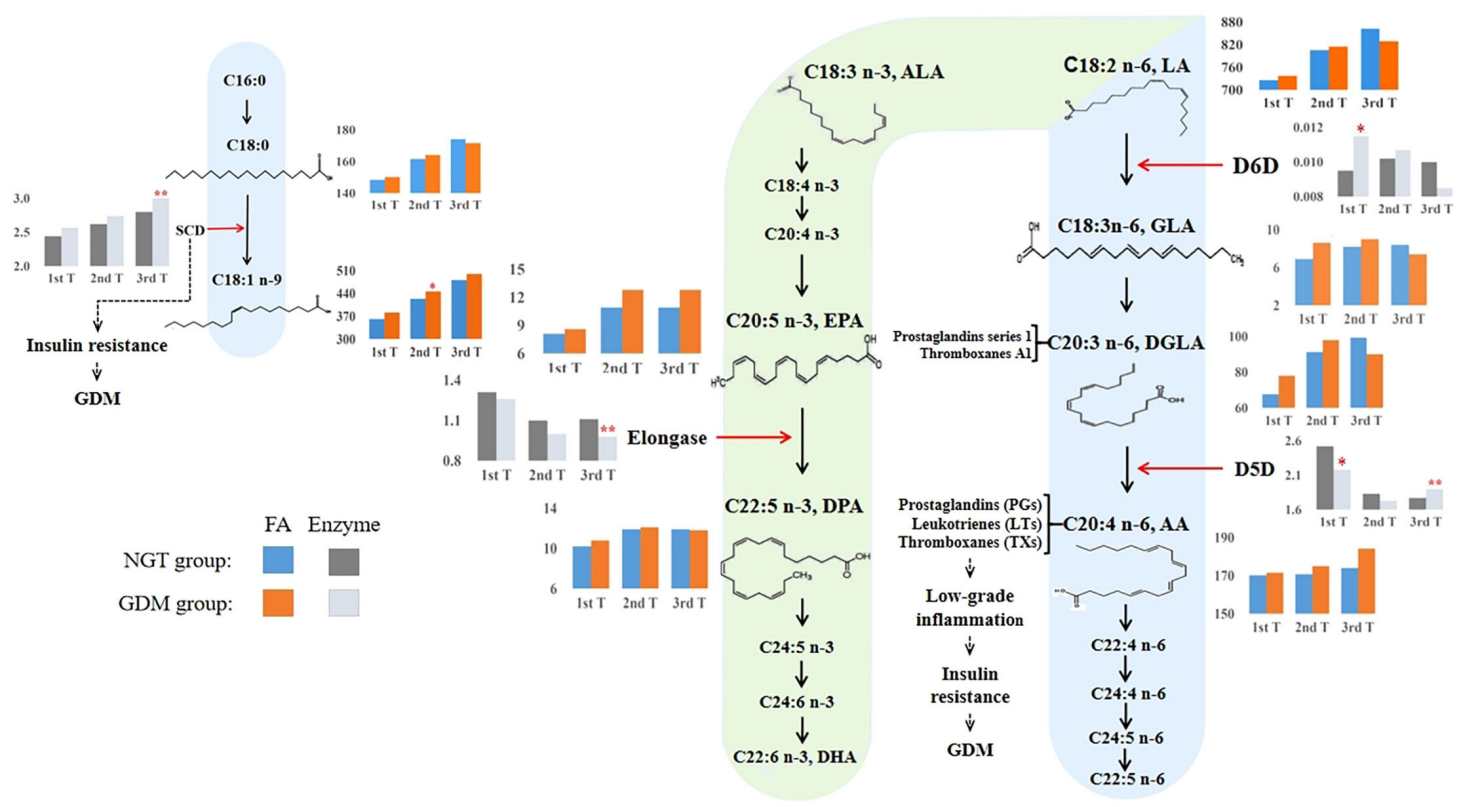
Overview of proposed GDM mechanisms based on how the long-chain fatty acid levels and estimated desaturase activities differed between GDM and NGT participants across trimesters. The n-3 and n-6 PUFA metabolic pathways (20, 38, 39) illustrated how high estimated D6D activity and low estimated D5D activity contributed to the early development of GDM. Conversely, higher estimated D5D activity was associated with a higher risk of GDM in the third trimester of pregnancy. The estimated desaturase activities of the maternal serum indicated a dysregulation of PUFA. In particular, the level of AA was increased throughout pregnancy in the GDM group. AA, as a precursor of pro-inflammatory eicosanoids (e.g., prostaglandins, thromboxanes, and leukotrienes), was associated with insulin resistance. Moreover, we also found that estimated SCD (delta-9–18 desaturase) activity increased significantly during pregnancy in women with GDM, promoting insulin resistance. Based on these observations, we hypothesize that dyshomeostasis of PUFAs caused by impaired D6D, D5D, and SCD activities may contribute to low-grade inflammation, subsequently advancing insulin resistance and the pathophysiology of GDM. 1st T, first trimester, 11–14 weeks; 2nd T, second trimester, 22–28 weeks; 3rd T, third trimester, 32–34 weeks; GDM, gestational diabetes mellitus; NGT, normal glucose tolerance; FA, fatty acid; C18:0, octadecanoic acid; C18:1n-9, octadecenoic acid; C18:2n-6, linoleic acid (LA); GLA, γ-linolenic acid; DGLA, eicosatrienoic acid; AA, arachidonic acid; C18:3n-3, α-linolenic acid; EPA, eicosapentaenoic acid; DPA, docosapentaenoic acid; SCD (stearoyl-CoA desaturase), C18:1n-9/C18:0; D6D, delta-6 desaturase, C18:3n-6/C18:2n-6; D5D, delta-5 desaturase, C20:4n−6/C20:3n−6; elongase, C22:5n-3/C20:5n-3. **: *p, q* < 0.01; *: *p, q* < 0.05. Blue and orange bars indicate the absolute concentrations of fatty acids in the NGT and GDM groups, respectively. Light black and gray bars indicate the desaturase activities for the NGT and GDM groups, respectively.

The different PUFA enzymatic activities in the n-3 and n-6 metabolic pathways between early and late pregnancy, shortlisted using the Mann–Whitney *U*-test with FDR and logistic regression adjusting for confounders, are displayed in Figure 4. During the first trimester, only the n-6 metabolic enzymes, D5D and D6D, showed significantly lower and higher enzymatic activities, respectively, in women with GDM than in the NGT group. Conversely, we found that only D5D was upregulated in the n-6 metabolic pathway and elongase was downregulated in the n-3 metabolic pathway in late pregnancy, when we compared the GDM group to the NGT group.

## Discussion

The present study investigated the association of the individual serum PUFA level and desaturase with GDM in early to late pregnancy. Totally, five fatty acid desaturase activities were assessed longitudinally, based on their product-to-precursor ratios, in a cohort of 661 pregnant women. Disparities in enzymatic activities between women with GDM and those with NGT were observed across pregnancy. In particular, estimated higher D6D and lower D5D activities were observed in women with GDM in the first trimester, while both desaturases exhibited inverse enzymatic activities in the third trimester. Discrepancies in D6D and D5D activities could give rise to abnormal levels of n-6 PUFAs (40), including linoleic acid (LA), GLA, DGLA, and AA which, in turn, could contribute to the development of insulin resistance and GDM.

### Physiological Changes in PUFA Profile Throughout Pregnancy

During gestation, physiological adaptations contribute to changes in the PUFA profile of healthy, pregnant women (41, 42). An overall gradual increase in the levels of PUFAs was observed from the first to the third trimester, irrespective of GDM. Initially, there is an anabolic stage with an elevation in lipogenesis and fat storage in preparation for the surge in fetal energy demands in the third trimester. During late pregnancy, lipid metabolism transitions to a net lipolysis with a catabolism of fat deposits (41, 42). These changes in lipid physiology across pregnancy ensure appropriate nutrient availability for the different stages of fetal development (10). The consumption of high-fat products has increased in recent years in China (43). Furthermore, the traditional Chinese diet includes eggs and meat feature prominently and provide a good source of essential fatty acids, high-quality protein, and micronutrients (44). Pregnant Chinese women are often encouraged to consume animal-derived foods more frequently (44). In combination, these factors contribute to a significant increase in dietary fat intake during pregnancy, predicted to aggravate hyperlipidemia and further increase the levels of PUFAs. A study has demonstrated that there was a persistent state of high concentrations of PUFAs during pregnancy from the first to the third trimester (45) and that elevated FFA levels are associated with inflammation-related metabolic diseases such as obesity, β-cell dysfunction, insulin resistance, and metabolic syndrome (46, 47). Thus, the changes of serum PUFAs influenced by dietary intake and lipid physiological adaptation in pregnancy are potentially highly relevant to the rising incidence of GDM in the Chinese population (44).

### Role of Arachidonic Acid in Pregnancy

Interestingly, elevated AA seems to be associated with insulin resistance *via* low-grade inflammation. AA is the product of D5D, and physiological concentrations of AA can enhance the influx of Ca^2+^ into β-cells by opening the Ca^2+^ channel, which increases Ca^2+^-dependent physiological processes such as protein kinase C (PKC) activity and exocytosis (48, 49). In turn, this promotes β-cells to produce and secrete insulin. However, a high concentration of AA inhibits Ca^2+^ influx and leads to the reduction of insulin secretion (48–50). Inflammatory mediators [such as prostaglandin E2, 15-hydroxyeicosatetraenoic acid (15-HETE), and LTs], produced by AA metabolism, reduce the insulin sensitivity of target tissues (liver, muscle, or adipose tissues) through the reduction in insulin receptor substrate 1 tyrosine phosphorylation and interference with insulin receptor signaling (9, 19, 51). It is important to note that GDM is accompanied by low-grade systemic inflammation, rather than acute inflammation, which could lead to other pregnancy complications, such as miscarriages and premature birth. AA is the main precursor of pro-inflammatory eicosanoids. PGs increase the likelihood of vaginal delivery within 24 h, but they can also stimulate the uterus to contract too much, leading to miscarriages (17). Higher levels of 5-HETE and 15-HETE are associated with premature delivery (52). Since our study only included GDM women delivered near term birth, it is accepted that we observed an upward trend of AA across gestation in GDM without excessive inflammatory activation. Moreover, studies with D5D activity-deficient mice have shown that selective D5D inhibitors improved insulin sensitivity and glycemic control (53, 54). Based on our results, the lower activity of D5D and lower level of AA were associated with a reduced risk of GDM. These findings had a potential therapeutic intervention for women with GDM, although further analyses are required.

### Estimated Fatty Acid Desaturase Activities Across GDM Gestation

In the first trimester of pregnancy, we found estimated D6D upregulation and D5D downregulation, which in combination would regulate the physiological level of PUFAs. This could be a protective metabolic mechanism against GDM pathophysiology. In the NGT group, we found that estimated D6D activity was downregulated in the first trimester of pregnancy, while estimated D5D activity was upregulated, and the levels of LA, GLA, DGLA, and AA were reduced compared to those women in the GDM group (Figure 4). Thus, in order to meet AA and insulin requirements, the body could adaptively upregulate the activity of D5D. On the other hand, we found that estimated D6D activity was upregulated, and estimated D5D activity was downregulated in the GDM group, with elevated levels of n-6 PUFAs such as LA, GLA, DGLA, and AA (Figure 4). Other longitudinal studies have reported that high D6D activity and low D5D activity are positively associated with several metabolic disorders, including T2D, obesity, and hypertension (25, 55, 56). Some studies have also reported that the concurrence of D5D and D6D activities with T2D incidence is dependent on plasma apoB-lipoprotein, which can be depleted by n-3 PUFAs (57, 58). We observed a higher essential n-6 LA concentration and a higher metabolism of its downstream D6D activity in GDM women in the first trimester. Moreover, studies have consistently reported that D5D activity was reduced in women with GDM between 10 and 14 gestational weeks (33, 59). Therefore, we speculate that reduced D5D activity in women with GDM is an adaptive process to reduce high levels of AA in order to combat subclinical inflammation in the first trimester as an early protective mechanism to minimize the risk of insulin resistance.

In the second trimester of pregnancy, when glucose and lipid metabolism was further dysregulated, the concentration of AA exceeded the physiological regulation capacity. In the GDM group, we found that the concentrations of the n-6 PUFAs, LA, GLA, DGLA, and AA, and the estimated activity of D6D were higher than in the NGT group (Figure 4). However, there was a slight decline in the estimated activity of D5D. Thus, we hypothesize that women with GDM cannot normally decrease the activity of D5D to inhibit the increase in the AA concentration in the second trimester of pregnancy. At this stage of pregnancy, irreversible pathophysiological changes related to inflammation such as insulin resistance, caused by abnormal AA levels, can begin to occur. The persistence of insulin resistance over time eventually results in the imbalance of glucose and lipid metabolism, and two main inflammatory pathways are activated, which can promote the development of GDM (60).

Last, in the physiological inflammatory response during late pregnancy, the concentrations of pro-inflammatory eicosanoids gradually increase in preparation for maternal parturition. Together with the low-grade pathological inflammation of GDM and worsened insulin resistance, lipid metabolism becomes abnormal in late pregnancy. We found that PUFA concentrations increased considerably toward the end of pregnancy in the third trimester. In the NGT group, we found that estimated D6D activity was upregulated, which would accelerate the metabolic degradation of LA. This is associated with increased concentrations of GLA and DGLA in the NGT group (Figure 4). Meanwhile, the activity of estimated D5D was downregulated, thereby reducing the concentration of AA to reach the physiological regulation capacity (Figure 4). A previous study of 21 diabetes-free pregnant women also found that levels of D5D activity in the third trimester of pregnancy was downregulated (24). Furthermore, disordered lipid metabolism associated with GDM was observed in the third trimester. Indeed, discrepancies in D6D and D5D activities were observed in the n-6 metabolic pathway in late pregnancy. Our results suggest that estimated D6D activity may be downregulated in the GDM group to disrupt lipid metabolism, leading to decreased GLA and DGLA concentrations (Figure 4). GLA exerts its anti-inflammatory activity through the formation of DGLA and downstream cyclooxygenase products, including prostaglandins of series 1 and thromboxane A1 (61). The downregulation of D6D activity could suppress the anti-inflammatory response by reducing the production of GLA. Furthermore, this is concomitant with D5D upregulation and excessive production of AA, potentially leading to an inflammatory phenotype and insulin resistance. In line with our findings, a prospective study of singleton pregnancies in Canada showed that GDM participants had higher D5D activity than non-GDM participants in the third trimester (32). Although D5D was upregulated in the n-6 metabolic pathway, elongase was downregulated in the n-3 metabolic pathway in late pregnancy of the GDM group (Figure 4). Elongase converts EPA to DPA, which in turn acts as a precursor for the biosynthesis of anti-inflammatory oxylipins such as protectins, maresins, and isoprostanes (39). It is interesting to speculate that the protective regulation of GLA, DGLA, AA, and DPA levels is disrupted in GDM and that the low D6D activity, the high D5D activity, and the low elongase activity may worsen GDM-associated phenotypes. Therefore, the lipid metabolic disorder observed in pregnant women with GDM might disrupt the synergistic regulation of pro-inflammatory and anti-inflammatory metabolic responses in the third trimester of pregnancy.

We also found that increased activation of delta-9–18 desaturase, which converts the saturated FA C18:0 to the mono-unsaturated FA C18:1n-9, is associated with the development of GDM in the third trimester. Our data also showed that estimated delta-9–18 desaturase activity increased significantly in the third trimester of pregnancy in women with GDM (Figure 4), and the concentration of its immediate product, octadecenoic acid (OA), was significantly increased in the second trimester (Figure 4). Studies have shown that obesity and insulin resistance are directly related to the activation or overexpression of delta-9–18 desaturase activity since delta-9–18 desaturase activity is increased by dietary glucose, fructose, and insulin (21, 23). A recent examination of OA in the adipose-like cell line (3T3 L1) showed that excess OA was associated with a higher lipid accumulation, as well as impaired insulin signaling and glucose utilization (62). Moreover, a rodent model has shown that insulin sensitivity was improved in delta-9–18 desaturase-deficient mice (63). Thus, the higher delta-9–18 desaturase activity observed in the third trimester was related to GDM.

### Strengths and Limitations

Our study has several strengths. It is the first prospective cohort study to investigate the association between fatty acid desaturase activities and GDM across the three trimesters of pregnancy, in addition to assessing fatty acid levels expressed as absolute concentrations. However, several limitations of this study also merit discussion. First, we did not measure fatty acid desaturase activities directly but evaluated them in terms of product-to-precursor ratios. Second, the participants diagnosed with GDM were managed with insulin treatment or food intervention, which may interfere with desaturase activities determined in the third trimester. However, this would not impact results obtained in the first trimester. Third, the maternal dietary questionnaire was not used to assess how maternal dietary habit affects serum free fatty acid levels. Last, although we have adjusted for several potential confounders, we could not fully exclude the possibility of other unexpected factors.

## Conclusion

In conclusion, we identified longitudinal changes in fatty acid concentrations and desaturase activities in GDM pregnancies. There were numerous fatty acid differences observed in each trimester in association with GDM. Higher D5D and delta-9–18 desaturase activities were associated with a higher risk of GDM. Our study adds important information to the current knowledge on GDM. We found that an imbalance in PUFA concentrations caused by abnormal desaturase activities may likely contribute to the insulin resistance of GDM. Hence, it is worth measuring these factors early in pregnancy to identify women at risk of high blood glucose and GDM. Future studies should consider the influences of dietary fatty acids and nutrient intakes on desaturase and elongase activities to better understand the risk of GDM.

## Data Availability Statement

The raw data supporting the conclusions of this article will be made available by the authors, without undue reservation.

## Ethics Statement

The studies involving human participants were reviewed and approved by Ethics Committee of Chongqing Medical University, China. The patients/participants provided their written informed consent to participate in this study. Written informed consent was obtained from the individual(s) for the publication of any potentially identifiable images or data included in this article.

## Author Contributions

T-LH and HZ were responsible for conceptualization and funding acquisition. YL and T-LH were responsible for methodology, software, and data analysis. TZ and YY were responsible for data collection. T-LH, RS, PB, HZ, and Y-YX were responsible for data curation. YL and Y-YX were responsible for writing the original draft. YL, TM, RS, and BN were responsible for data interpretation and manuscript editing. All authors read and approved the final manuscript.

## Funding

This work was supported by the National Natural Science Foundation of China (Nos. 81971406, 81771607, 81871185, and 81901507), 111 Project [Yuwaizhuan (2016)32], Future Medicine Youth Innovation Team of CQMU (No. W0083), Kuanren Talents Programs, Chongqing Municipal Education Commission (KJZD-K202100407), Chongqing Health Commission and Chongqing Science & Technology Commission (2021MSXM121, cstc2021jcyj-msxmX0213, and 2020MSXM101), and Smart Medicine Research Project of Chongqing Medical University (No. ZHYX202103).

## Conflict of Interest

The authors declare that the research was conducted in the absence of any commercial or financial relationships that could be construed as a potential conflict of interest. The handling editor, DL declared a past collaboration with author, PB.

## Publisher's Note

All claims expressed in this article are solely those of the authors and do not necessarily represent those of their affiliated organizations, or those of the publisher, the editors and the reviewers. Any product that may be evaluated in this article, or claim that may be made by its manufacturer, is not guaranteed or endorsed by the publisher.

## Abbreviations

AA: arachidonic acid
BMI: body mass index
CLIMB: Complex Lipids in Mothers and Babies
DGLA: eicosatrienoic acid
DHA: docosahexaenoic acid
D5D: delta-5 desaturase
D6D: delta-6 desaturase
EPA: eicosapentaenoic acid
FA: fatty acid
FFAs: free fatty acids
FG: fasting blood glucose
GC-MS: gas chromatography–mass spectrometry
GDM: gestational diabetes mellitus
GLA: γ-linolenic acid
ICH-GCP: International Conference on Harmonization Good Clinical Practice
LA: linoleic acid
LTs: leukotrienes
MUFAs: monounsaturated fatty acids
NGT: normal glucose tolerance
OA: octadecenoic acid
OGTT: oral glucose tolerance test
OR: odds ratio
PGs: prostaglandins
PKC: protein kinase C
PUFA: polyunsaturated fatty acid
SCD: stearoyl-CoA desaturase
SFAs: saturated fatty acids
T2D: type 2 diabetes.
