# Supplementary material for: Complex Interactions Between Circulating Fatty Acid Levels, Desaturase Activities, and the Risk of Gestational Diabetes Mellitus: A Prospective Cohort Study
Source: Front Nutr. 2022 Jul 11;9:919357. doi: 10.3389/fnut.2022.919357 (PMC9313599; doi:10.3389/fnut.2022.919357)
Supplement: Supplementary file 1 [file Data_Sheet_1.docx]

| **SUPPLEMENTARY TABLE 1 │** Comparison of serum fatty acid concentrations (mg/L) and odds ratios in the first trimester between pregnant women with normal glucose tolerance (NGT) and gestational diabetes mellitus (GDM). | | | | | | |
| --- | --- | --- | --- | --- | --- | --- |
| Fatty acids | GDM (n=189) | NGT (n=472) | p^a^ value | q value | OR (95%CI) | *p^b^* value |
| Tetradecanoic acid | 12.1 (8.4, 17.4) | 10.4 (7.7, 15.4) | 0.01^*^ | 0.03^*^ | 1.28 (0.91, 1.78) | 0.15 |
| Hexadecanoic acid | 462.2 (389.7, 549.4) | 457.8 (389.9, 523.8) | 0.18 | 0.22 | 1.24 (0.56, 2.75) | 0.60 |
| Octadecanoic acid | 150.2 (131.4, 168.7) | 148.4 (130.6, 167.3) | 0.47 | 0.37 | 0.97 (0.42, 2.26) | 0.95 |
| Arachic acid | 6.0 (5.0, 7.0) | 5.9 (5.0, 6.9) | 0.67 | 0.44 | 0.98 (0.51, 1.87) | 0.94 |
| Docosanoic acid | 13.7 (11.2, 16.8) | 13.7 (11.6, 16.7) | 0.55 | 0.39 | 0.66 (0.37, 1.18) | 0.16 |
| Lignoceric acid | 9.8 (7.7, 12.5) | 10.2 (8.2, 12.6) | 0.10 | 0.15 | 0.61 (0.37, 1.02) | 0.06 |
| SFA | 656.8 (560.1, 764.5) | 647.2 (564.5, 740.3) | 0.25 | 0.24 | 1.16 (0.50, 2.71) | 0.73 |
| Hexadecenoic acid | 23.9 (17.9, 31.8) | 22.2 (16.8, 29.1) | 0.03^*^ | 0.06 | 1.28 (0.86, 1.91) | 0.23 |
| Octadecenoic acid | 381.4 (316.5, 451.5) | 361.4 (307.9, 424.5) | 0.02^*^ | 0.06 | 1.52 (0.73, 3.17) | 0.26 |
| Eicosaenoic acid | 5.0 (3.9, 6.7) | 4.7 (3.9, 6.3) | 0.21 | 0.24 | 1.19 (0.78, 1.84) | 0.42 |
| Tetracosenic acid | 34.4 (25.6, 42.0) | 33.4 (27.2, 42.6) | 0.86 | 0.51 | 0.94 (0.57, 1.55) | 0.80 |
| MUFA | 450.0 (364.8, 530.3) | 426.3 (363.2, 496.4) | 0.03^*^ | 0.06 | 1.50 (0.72, 3.12) | 0.28 |
| α-linolenic acid | 28.6 (21.2, 39.5) | 25.9 (19.3, 33.2) | 0.01^*^ | 0.02^*^ | 1.56 (1.03, 2.36) | 0.04^*^ |
| Eicosapentaenoic acid | 8.6 (6.0, 14.1) | 8.1 (5.2, 13.2) | 0.11 | 0.15 | 1.10 (0.84, 1.44) | 0.49 |
| Docosapentenoic acid | 10.8 (8.7, 14.6) | 10.2 (8.6, 13.0) | 0.07 | 0.10 | 1.22 (0.73, 2.03) | 0.45 |
| Docosahexaenoic acid | 69.9 (56.4, 81.7) | 65.6 (55.6, 81.6) | 0.28 | 0.24 | 1.19 (0.68, 2.07) | 0.54 |
| ∑n-3LCPUFA | 119.0 (98.9, 146.5) | 113.6 (94.6, 138.9) | 0.04^*^ | 0.06 | 1.46 (0.81, 2.63) | 0.21 |
| Linoleic acid | 736.9 (618.2, 833.9) | 726.0 (641.9, 838.0) | 0.86 | 0.51 | 0.81 (0.36, 1.83) | 0.61 |
| γ-linolenic acid | 8.6 (5.8, 11.6) | 6.9 (4.7, 10.2) | ＜0.001^***^ | ＜0.001^***^ | 1.35 (0.99, 1.83) | 0.06 |
| Eicosadienoic acid | 9.5 (7.6, 11.7) | 9.3 (7.5, 11.6) | 0.28 | 0.24 | 1.10 (0.65, 1.89) | 0.72 |
| Arachidonic acid | 171.5 (142.4, 209.2) | 170.2 (138.9, 202.4) | 0.50 | 0.38 | 1.04 (0.56, 1.92) | 0.90 |
| Eicosatrienoic acid | 78.0 (58.6, 102.5) | 67.7 (52.5, 87.9) | 0.001^**^ | 0.007^**^ | 1.55 (0.98, 2.45) | 0.06 |
| Docosatetraenoic acid | 6.2 (5.4, 7.7) | 6.1 (4.9, 7.5) | 0.26 | 0.24 | 1.08 (0.61, 1.92) | 0.79 |
| ∑n-6LCPUFA | 1011.3 (873.4, 1150.9) | 1004.2 (892.0,1128.9) | 0.57 | 0.39 | 0.97 (0.41, 2.27) | 0.94 |
| *Data are presented as medians (25th percentile, 75th percentile) for Mann-Whitney test; the data have been ln-transformed for binary logistic regression* | | | | | | |
| *Binary logistic regression analyses were adjusted for age, BMI, smoking and drinking, primiparous* | | | | | | |
| p^a^ *values are for statistical comparison between the two groups (Mann-Whitney test)* | | | | | | |
| *p^b^ values are for odds ratio between the two groups (binary logistic regression)* | | | | | | |
| *The q values (adjusted p-values) were performed by false discovery rate (FDR)* | | | | | | |
| *p^a,b^ values and q values are significant at p, q <0.05; ***p, q < 0.001, **p, q < 0.01, *p, q < 0.05* | | | | | | |
| *NGT-normal glucose tolerance; GDM-gestational diabetes mellitus; SFA-saturated fatty acids; MUFA-monounsaturated fatty acids; LCPUFA-long chain polyunsaturated fatty acid; OR-odds ratio; CI-confidence intervals* | | | | | | |

| **SUPPLEMENTARY TABLE 2 │** Comparison of serum fatty acid concentrations (mg/L) and odds ratios in the second trimester between pregnant women with normal glucose tolerance (NGT) and gestational diabetes mellitus (GDM). | | | | | | |
| --- | --- | --- | --- | --- | --- | --- |
| Fatty acids | GDM (n=189) | NGT (n=472) | p^a^ value | q value | OR (95%CI) | p^b^ value |
| Tetradecanoic acid | 20.9 (14.6, 30.5) | 20.0 (14.8, 28.5) | 0.46 | 0.39 | 1.10 (0.76, 1.57) | 0.62 |
| Hexadecanoic acid | 565.5 (490.9, 670.1) | 551.3 (479.4, 635.5) | 0.13 | 0.22 | 1.62 (0.75, 3.51) | 0.22 |
| Octadecanoic acid | 164.2 (145.0, 187.8) | 161.6 (144.7, 181.4) | 0.20 | 0.22 | 1.56 (0.64, 3.79) | 0.33 |
| Arachic acid | 6.6 (5.7, 7.9) | 6.7 (5.8, 7.9) | 0.90 | 0.56 | 0.97 (0.50, 1.90) | 0.94 |
| Docosanoic acid | 15.2 (13.1, 18.9) | 16.2 (13.7, 19.1) | 0.03^*^ | 0.07 | 0.55 (0.30, 1.00) | 0.05 |
| Lignoceric acid | 10.7 (8.6, 13.5) | 11.9 (9.5, 14.6) | 0.002^**^ | 0.03^*^ | 0.53 (0.32, 0.87) | 0.01^*^ |
| SFA | 790.6 (701.4, 913.9) | 764.0 (680.7, 879.5) | 0.14 | 0.22 | 1.61 (0.69, 3.76) | 0.27 |
| Hexadecenoic acid | 31.7 (21.6, 46.8) | 30.0 (22.5, 43.8) | 0.54 | 0.41 | 1.10 (0.77, 1.57) | 0.60 |
| Octadecenoic acid | 446.5 (372.6, 523.9) | 423.7 (364.8, 491.3) | 0.01^*^ | 0.04^*^ | 2.48 (1.16, 5.28) | 0.02^*^ |
| Eicosaenoic acid | 6.5 (5.1, 8.2) | 6.1 (4.9, 8.2) | 0.18 | 0.22 | 1.13 (0.75, 1.71) | 0.57 |
| Tetracosenic acid | 38.4 (31.2, 48.4) | 38.8 (31.0, 47.9) | 0.89 | 0.56 | 1.04 (0.64, 1.69) | 0.86 |
| MUFA | 527.2 (447.8, 643.1) | 502.7 (436.2, 588.7) | 0.02^*^ | 0.05 | 2.21 (1.04, 4.68) | 0.04^*^ |
| α-linolenic acid | 40.7 (29.0, 51.4) | 35.7 (26.5, 48.2) | 0.01^*^ | 0.05 | 1.69 (1.14, 2.50) | 0.008^* *^ |
| Eicosapentaenoic acid | 12.8 (8.0, 17.7) | 10.9 (7.1, 16.5) | 0.03^*^ | 0.07 | 1.32 (1.00, 1.74) | 0.05 |
| Docosapentenoic acid | 12.1 (9.5, 15.7) | 11.9 (9.3, 15.0) | 0.41 | 0.37 | 1.25 (0.76, 2.03) | 0.38 |
| Docosahexaenoic acid | 83.3 (66.3, 98.5) | 81.2 (64.6, 100.2) | 0.62 | 0.45 | 1.12 (0.66, 1.91) | 0.67 |
| ∑n-3LCPUFA | 151.0 (120.7, 187.3) | 142.6 (119.8, 173.9) | 0.09 | 0.18 | 1.67 (0.93, 2.99) | 0.09 |
| Linoleic acid | 814.8 (695.2, 930.5) | 805.7 (699.8, 920.9) | 0.93 | 0.56 | 1.05 (0.45, 2.47) | 0.91 |
| γ-linolenic acid | 9.0 (6.2, 12.8) | 8.2 (5.7, 12.5) | 0.16 | 0.22 | 1.23 (0.90, 1.67) | 0.19 |
| Eicosadienoic acid | 11.9 (9.6, 14.4) | 12.0 (10.0, 14.6) | 0.33 | 0.34 | 0.69 (0.37, 1.26) | 0.22 |
| Arachidonic acid | 175.0 (147.9, 202.8) | 170.7 (142.5, 204.8) | 0.36 | 0.34 | 1.23 (0.66, 2.30) | 0.52 |
| Eicosatrienoic acid | 98.0 (72.8, 120.6) | 91.3 (72.4, 116.1) | 0.20 | 0.22 | 1.19 (0.73, 1.95) | 0.49 |
| Docosatetraenoic acid | 7.0 (5.8, 8.6) | 7.1 (5.9, 8.6) | 0.92 | 0.56 | 0.97 (0.55, 1.71) | 0.91 |
| ∑n-6LCPUFA | 1130.6 (991.9, 1251.4) | 1113.6 (972.4, 1243.3) | 0.55 | 0.41 | 1.16 (0.47, 2.89) | 0.75 |
| *Data are presented as medians (25th percentile, 75th percentile) for Mann-Whitney test; The data have been ln-transformed for binary logistic regression* | | | | | | |
| *Binary logistic regression analyses were adjusted for age, BMI, smoking and drinking, primiparous* | | | | | | |
| *p^a^ values are for statistical comparison between the two groups (Mann-Whitney test)* | | | | | | |
| *p^b^ values are for odds ratio between the two groups (binary logistic regression)* | | | | | | |
| *The q values (adjusted p-values) were performed by false discovery rate (FDR)* | | | | | | |
| *p^a,b^ values and q values are significant at p, q <0.05; ***p, q < 0.001, **p, q < 0.01, *p, q < 0.05* | | | | | | |
| *NGT-normal glucose tolerance; GDM-gestational diabetes mellitus; SFA-saturated fatty acids; MUFA-monounsaturated fatty acids; LCPUFA-long chain polyunsaturated fatty acid; OR-odds ratio; CI-confidence intervals* | | | | | | |

| **SUPPLEMENTARY TABLE 3 │** Comparison of serum fatty acid concentrations (mg/L) and odds ratios in the third trimester between pregnant women with normal glucose tolerance (NGT) and gestational diabetes mellitus (GDM). | | | | | | |
| --- | --- | --- | --- | --- | --- | --- |
| Fatty acids | GDM (n=189) | NGT (n=472) | p^a^ value | q value | OR (95%CI) | P^b^ value |
| Tetradecanoic acid | 21.8 (15.0, 31.4) | 23.0 (16.7, 31.5) | 0.16 | 0.19 | 0.75 (0.53, 1.08) | 0.13 |
| Hexadecanoic acid | 627.2 (525.8, 709.2) | 625.4 (525.7, 718.7) | 0.84 | 0.53 | 0.85 (0.40, 1.80) | 0.68 |
| Octadecanoic acid | 171.6 (151.0, 190.3) | 174.1 (152.0, 196.3) | 0.37 | 0.28 | 0.63 (0.26, 1.53) | 0.30 |
| Arachic acid | 7.1 (6.1, 8.3) | 7.1 (6.2, 8.4) | 0.41 | 0.30 | 0.70 (0.36, 1.37) | 0.30 |
| Docosanoic acid | 15.4 (12.9, 18.2) | 16.8 (14.4, 20.1) | ＜0.001*** | ＜0.001*** | 0.36 (0.19, 0.65) | 0.001^**^ |
| Lignoceric acid | 10.2 (8.3, 13.1) | 11.6 (9.5, 14.5) | ＜0.001*** | ＜0.001*** | 0.37 (0.22, 0.62) | <0.001^***^ |
| SFA | 856.2 (738.5, 952.6) | 864.4 (748.1, 977.7) | 0.5 | 0.35 | 0.72 (0.31, 1.64) | 0.43 |
| Hexadecenoic acid | 32.2 (25.2, 44.5) | 35.9 (26.5, 50.4) | 0.04* | 0.09 | 0.69 (0.47, 1.03) | 0.07 |
| Octadecenoic acid | 500.0 (421.0, 579.3) | 480.7 (415.7, 563.5) | 0.04* | 0.11 | 1.79 (0.85, 3.78) | 0.13 |
| Eicosaenoic acid | 7.6 (5.8, 10.3) | 7.2 (5.5, 9.4) | 0.09 | 0.14 | 1.41 (0.94, 2.10) | 0.10 |
| Tetracosenic acid | 41.2 (32.6, 50.8) | 41.1 (33.0, 51.4) | 0.66 | 0.43 | 0.84 (0.53, 1.33) | 0.45 |
| MUFA | 594.3 (501.2, 685.3) | 575.9 (486.4, 665.2) | 0.19 | 0.21 | 1.47 (0.69, 3.13) | 0.32 |
| α-linolenic acid | 41.9 (30.8, 56.3) | 40.9 (29.8, 54.1) | 0.27 | 0.25 | 1.35 (0.91, 1.98) | 0.13 |
| Eicosapentaenoic acid | 12.8 (8.0, 18.6) | 10.9 (7.1, 16.7) | 0.03* | 0.09 | 1.33 (1.00, 1.76) | 0.05 |
| Docosapentenoic acid | 11.8 (9.6, 14.6) | 11.9 (9.7, 15.0) | 0.97 | 0.58 | 0.93 (0.56, 1.53) | 0.77 |
| Docosahexaenoic acid | 89.4 (71.2, 105.5) | 84.3 (68.5, 106.4) | 0.35 | 0.28 | 1.14 (0.66, 1.97) | 0.63 |
| ∑n-3LCPUFA | 163.4 (133.3, 186.9) | 152.3 (125.2, 183.7) | 0.11 | 0.16 | 1.50 (0.83, 2.69) | 0.18 |
| Linoleic acid | 829.6 (698.0, 973.7) | 862.5 (733.9, 967.4) | 0.23 | 0.22 | 0.62 (0.28, 1.40) | 0.25 |
| γ-linolenic acid | 7.4 (5.2, 11.2) | 8.4 (5.9, 12.3) | 0.04* | 0.09 | 0.73 (0.53, 1.00) | 0.05 |
| Eicosadienoic acid | 12.0 (9.6, 15.0) | 12.6 (10.3, 15.3) | 0.06 | 0.11 | 0.58 (0.32, 1.04) | 0.07 |
| Arachidonic acid | 184.2 (151.2, 209.3) | 174.1 (144.3, 209.3) | 0.32 | 0.27 | 1.15 (0.62, 2.13) | 0.67 |
| Eicosatrienoic acid | 90.1 (71.8, 118.8) | 99.4 (77.6, 125.6) | 0.02* | 0.09 | 0.55 (0.33, 0.90) | 0.02* |
| Docosatetraenoic acid | 7.0 (5.8, 8.6) | 7.3 (5.9,9.0) | 0.12 | 0.17 | 0.61 (0.35, 1.08) | 0.09 |
| ∑n-6LCPUFA | 1134.9 (986.8, 1297.0) | 1174.6 (1027.5, 1304.9) | 0.22 | 0.22 | 0.57 (0.24, 1.36) | 0.21 |
| *Data are presented as medians (25th percentile, 75th percentile) for Mann-Whitney test; the data have been ln-transformed for binary logistic regression* | | | | | | |
| *Binary logistic regression analyses were adjusted for age, BMI, smoking and drinking, primiparous* | | | | | | |
| *p*a *values are for statistical comparison between the two groups (Mann-Whitney test)* | | | | | | |
| *p*b *values are for odds ratio between the two groups (binary logistic regression)* | | | | | | |
| *The q values (adjusted p-values) were performed by false discovery rate (FDR)* | | | | | | |
| *p^a,b^ values and q values are significant at p, q <0.05; ***p, q < 0.001, **p, q < 0.01, *p, q < 0.05* | | | | | | |
| *NGT-normal glucose tolerance; GDM-gestational diabetes mellitus; SFA-saturated fatty acids; MUFA-monounsaturated fatty acids; LCPUFA-long chain polyunsaturated fatty acid; OR-odds ratio; CI-confidence intervals* | | | | | | |

| **SUPPLEMENTARY TABLE 4 │** The interaction of desaturase activity between GDM and three-time points in either the mixed model with confounders or without confounders. | | |
| --- | --- | --- |
|  | **q-value** | **q-value (adjusted by age, BMI and primiparous)** |
| Delta-5 desaturase [C20:4 n–6/C20:3 n–6] | < 0.01 | < 0.01 |
| Delta-6 desaturase [C18:3 n–6/C18:2 n–6] | < 0.01 | < 0.01 |
| Delta-9-16 desaturase [C16:1 n–7/C16:0] | < 0.01 | < 0.01 |
| Delta-9-18 desaturase [C18:1 n-9/C18:0] | 0.56 | 0.54 |
| Elongase [C22:5 n-3/C20:5 n-3] | 0.21 | 0.20 |
| *BMI: body mass index* |  |  |

| **SUPPLEMENTARY TABLE 5** │ Standard and internal standard substance information. | | | | |
| --- | --- | --- | --- | --- |
| FAME standards* | Fatty acids | Manufacturer | Purity | Batch number |
| Methyl myristate | Tetradecanoic acid (C14:0) | Sigma | ≥99% | Lot#071M1509V |
| Methyl palmitate | Hexadecanoic acid (C16:0) | Sigma | ≥99% | Lot#SLBG4424V |
| Methyl palmitoleate | Hexadecenoic acid (C16:1 n-7) | Sigma | ≥99% | Lot#SLBB1046V |
| Heptadecanoic acid | Heptadecanoic acid (C17:0) | Sigma | ≥99% | Lot 099K1181 |
| Methyl stearate | Octadecanoic acid (C18:0) | Sigma | ≥99% | Lot#BCBK6253V |
| Methyl oleate | Octadecenoic acid (C18:l n-9) | Sigma | ≥99% | Lot#MKBL9030V |
| Methyl linoleate | Linoleic acid (C18:2 n-6) | Sigma | ≥99% | Lot#BCBG7530V |
| Methyl-γ-linolenate | γ-Linolenic acid (C18:3 n-6) | Sigma | ≥99% | Lot#SLBD0092V |
| α-Methyl linolenate | α-Linolenic acid (C18:3 n-3) | Sigma | ≥99% | Lot#SLBD5909V |
| Methyl arachidate | Arachidic acid (C20:0) | Sigma | ≥99% | Lot#SLBD2753V |
| Methyl cis-11-eicosenoate | Eicosenoic acid (C20:l n-9) | Sigma | ≥99% | Lot#SLBF1161V |
| cis-11,14-Eicosadienoic acid, methyl ester | Eicosadienoic acid（C20:2 n-6） | Sigma | ≥99% | 1G010010 |
| 8,11,14-Eicosatfienoic acid, methyl ester | Eicosatrienoic acid (C20:3 n-6) | Sigma | ≥99% | 4G010010 |
| cis-5,8,11,14-Eicosatetraenoic acid, methyl ester | Arachidonic acid (C20:4 n-6) | Sigma | ≥99% | Lot#SLBC5923V |
| Docosanoic acid, methyl ester | Docosanoic acid (C22:0) | Sigma | ≥99% | Lot#80207 |
| Methyl all-cis-5,8,11,14,17-eicosapentaenoate | Eicosapentaenoic acid (C20:5 n-3) | Sigma | ≥99% | Lot#BCBK2894V |
| cis-7,10,13,16-Docosatetranoic acid, methyl ester | Docosatetraenoic acid (C22:4 n-6) | Sigma | ≥99% | Lot#SLBG5166V |
| Tetracosanoic acid, methyl ester | Lignoceric acid (C24:0) | Sigma | ≥99% | Lot#00818 |
| Methyl all-cis-7,10,13,16,19-docosapentaenoate | Docosapentenoic acid (C22:5 n-3) | Sigma | ≥99% | Lot#BCBF6777V |
| Methyl 4,7,10,13,16,19-docosahexaenoate | Docosahexaenoic acid (C22:6 n-3) | Sigma | ≥99% | Lot#BCBL9396V |
| Methyl cis-15-tetracosenoate | Tetracosenoic acid (C24:1 n-9) | Sigma | ≥99% | Lot#BCBK2334 |
| *Abbreviation: FAME, fatty acid methyl ester* | | | | |


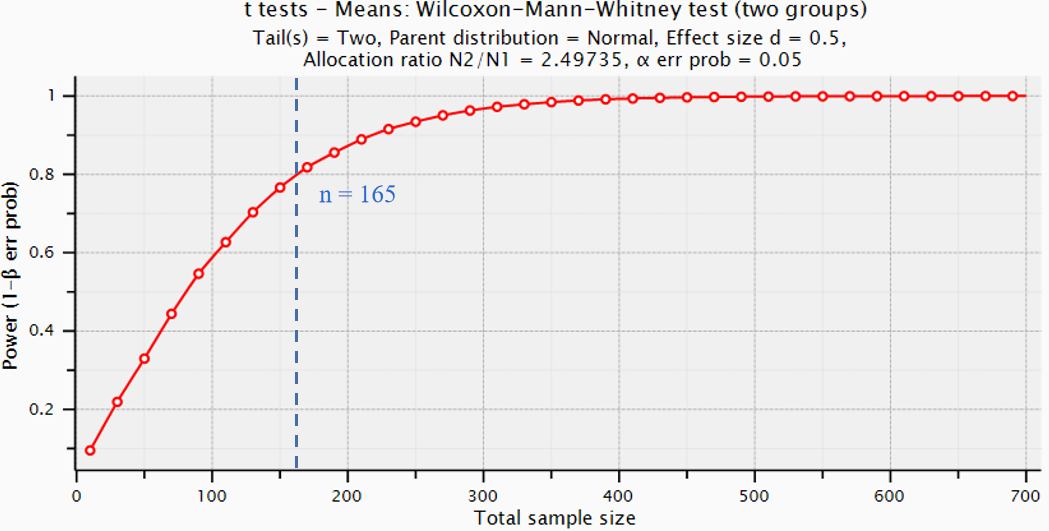


**SUPPLEMENTARY FIGURE 1│** The post hoc power analysis of sample sizes between GDM and control women at pregnancy for the Wilcoxon-Mann-Whitney test. The curve displays the relationship between changes in power (y-axis) with changes in total sample size (x-axis). The blue vertical line indicates the sample size required to have 80% power with an alpha value less than 0.05 for a given mean difference between the two groups. n= number, GDM = gestational diabetes mellitus.


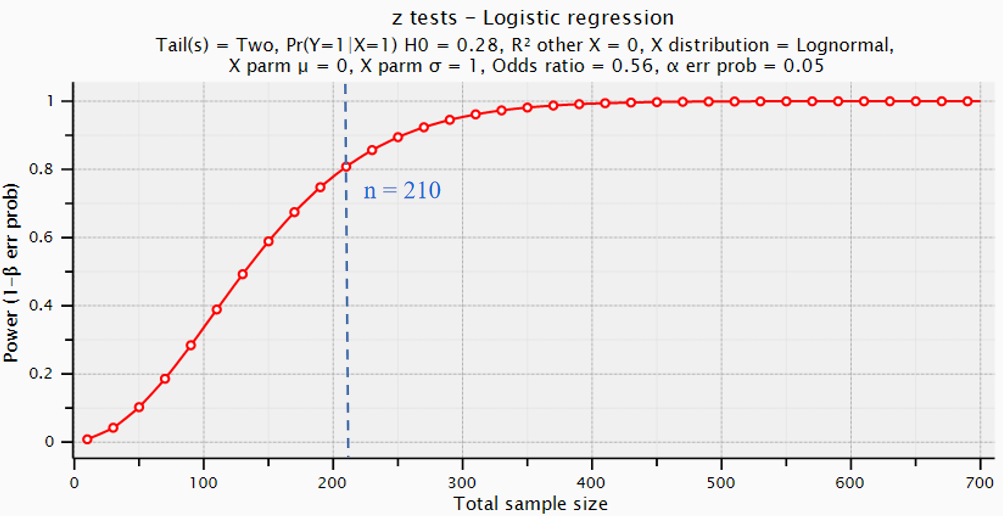


**SUPPLEMENTARY FIGURE 2│**The post hoc power analysis of sample sizes between GDM and control women at pregnancy for logistic regression. The curve displays the relationship between changes in power (y-axis) with changes in total sample size (x-axis). The blue vertical line indicates the sample size required to have 80% power with an alpha value less than 0.05 for a given mean difference between the two groups. n= number, GDM = gestational diabetes mellitus.
